# Predicted vs. Observed Prosthesis–Patient Mismatch After Surgical Aortic Valve Replacement

**DOI:** 10.3390/medicina61040743

**Published:** 2025-04-17

**Authors:** Giorgia Cibin, Augusto D’Onofrio, Giulia Lorenzoni, Valentina Lombardi, Emma Bergonzoni, Assunta Fabozzo, Irene Cao, Andrea Francavilla, Chiara Tessari, Dario Gregori, Gino Gerosa

**Affiliations:** 1 Cardiac Surgery Unit, Azienda Ospedale Università di Padova, 35122 Padova, Italy; giorgia.cibin@aopd.veneto.it (G.C.); valentinalombardi15@gmail.com (V.L.); bergonzoni.emma@gmail.com (E.B.); assunta.fabozzo@aopd.veneto.it (A.F.); irene.cao@studenti.unipd.it (I.C.); chiara.tessari@unipd.it (C.T.); gino.gerosa@unipd.it (G.G.); 2 Cardiac Surgery Unit, Università degli Studi di Roma Tor Vergata, 00133 Roma, Italy; 3 Unit of Biostatistics, Epidemiology and Public Health, Department of Cardiac, Thoracic, Vascular Sciences and Public Health, University of Padova, 35122 Padova, Italy; giulia.lorenzoni@unipd.it (G.L.); andrea.francavilla@ubep.unipd.it (A.F.); dario.gregori@unipd.it (D.G.)

**Keywords:** aortic valve replacement, bioprostheses, prosthesis–patient mismatch

## Abstract

*Background and Objectives*: Prosthesis–patient mismatch (PPM) after surgical aortic valve replacement (SAVR) is associated with worse clinical outcomes and worse valve durability. The aim of this retrospective single-center study was to evaluate the consistency between predicted PPM (PPMp) and measured PPM (PPMm) after SAVR with three different bioprostheses. *Materials and Methods*: We analyzed data of all consecutive patients who underwent surgical aortic valve replacement with Magna Ease, Intuity, and Inspiris Resilia bioprostheses (Edwards Lifesciences, Irvine, CA, USA) at our institution. PPM was defined if EOAi ≤ 0.85 cm^2^/m^2^. PPMm was determined by institutional echo lab-measured EOAi on discharge-day echocardiogram. PPMp was assessed using reference values for each valve model and size indexed to BSA based on height, weight, prosthesis type, and size. For the overall population and for the three valve types we evaluated the sensitivity, specificity, positive predicted value, negative predicted value, and accuracy of PPMp. Furthermore, the consistency between PPMm and PPMp were evaluated according to prosthesis type, size, stent internal diameter (ID), and true ID. *Results*: A total of 1323 patients underwent SAVR; complete hemodynamic data were available for 872 patients, who represent the population of our study. Magna Ease, Intuity, and Inspiris Resilia were implanted in 446 (51.1%), 341 (39.1%), and 85 (9.7%) patients, respectively. In 635 out of 872 cases (72.8%), PPMp was consistent with PPMm (Magna Ease: 321/446, 72%; Inspiris Resilia: 58/85, 68.2%; Intuity: 256/341, 75%). Overall, the sensitivity, specificity, positive predicted value, negative predicted value, and accuracy of PPMp were 0.26, 0.83, 0.24, 0.84, and 0.73, respectively (Magna Ease: 0.21, 0.82, 0.3, 0.8, and 0.72; Inspiris Resilia: 0.11, 0.82, 0.14, 0.79, and 0.68; Intuity: 0.45, 0.78, 0.19, 0.93, and 0.75). *Conclusions*: The consistency between PPMp and PPMm was suboptimal. We did not observe differences between PPMp and PPMm among different valve types. Discordance between PPMp and PPMm was more evident in smaller valve sizes. When implanting small valves, the evaluation of PPMp should be used with caution to avoid unexpected PPMm.

## 1. Introduction

Surgical aortic valve replacement (SAVR) has served as the gold standard for millions of patients worldwide affected by a pathology of the aortic valve, especially in cases of severe aortic stenosis (AS) [1]. Since its introduction in 1967 [2], SAVR has served as the gold standard for millions of patients, and despite the rapid adoption of transcatheter modalities, SAVR remains the preferred option for young, low-risk patients due to its proven durability and well-established outcomes [3,4].

The advent of transcatheter aortic valve replacement (TAVR) in 2002 [5] has provided a minimally invasive alternative, initially reserved for high-risk patients [6] but now extended to include intermediate [7] and low-risk [8] candidates with old age.

One of the most discussed issues in recent years is the incidence of prosthesis–patient mismatch (PPM), an aspect that was described for the first time by Rahimtoola et al. in 1978 [9] and that is still the subject of debate today.

PPM occurs when the effective orifice area (EOA) of the prosthetic valve is insufficient relative to the patient’s body surface area (BSA), resulting in elevated postoperative gradients and adverse clinical outcomes. PPM has been linked to reduced valve durability, slower regression of left ventricular hypertrophy (LVH), and an increased incidence of cardiac events [10,11].

The occurrence of PPM is multifactorial, influenced by patient-specific characteristics, prosthesis design, surgical technique, and prevailing hemodynamic conditions [12]. Addressing PPM is critical, particularly in younger patients, to ensure optimal long-term outcomes. In this context, surgical techniques that minimize the risk of PPM, coupled with careful prosthesis selection, reinforce the enduring value of SAVR as the first-line treatment for young, low-risk patients.

The selection of the optimal intervention involves balancing the benefits of SAVR with the evolving capabilities of TAVR, emphasizing the importance of individualized patient assessment. Accurate preoperative prediction of PPM is essential for selecting the optimal valve size and type to minimize the risk of PPM.

This prediction can be accomplished by dividing the normal reference value of the EOA for the intended prosthesis model and size by the patient’s BSA. Additionally, tools such as the “Valve PPM” mobile application facilitate the prediction of PPM by incorporating patient measurements and EOA values for various prosthetic valves.

The aim of this study was to evaluate the incidence of predicted PPM (PPMp) versus measured PPM (PPMm) following SAVR with three different bioprostheses: Magna Ease, Intuity, and Inspiris Resilia.

Furthermore, the study aimed to assess the accuracy of PPM measurement and its consistency across these bioprosthetic valve types.

## 2. Materials and Methods

This is a retrospective, single-center study. We included 1323 consecutive patients who underwent surgical aortic valve replacement (SAVR) for aortic stenosis or regurgitation, isolated or combined with other procedures, at our institution during the study period (July 2016–July 2023). Patients received one of the three bioprosthetic valves under investigation: Magna Ease, Intuity, or Inspiris Resilia (Edwards Lifesciences, Irvine, CA, USA).

Exclusion criteria were aortic dissection, endocarditis, and patients who previously underwent cardiac surgery.

PPM is categorized as moderate (EOA: 0.65–0.85 cm^2^/m^2^) or severe (EOA < 0.65 cm^2^/m^2^). In this study, we considered only the presence or absence of PPM (EOAi of ≤0.85 cm^2^/m^2^).

The PPMm was determined by the institutional echocardiography laboratory using EOAi measurements from echocardiograms performed on the day of discharge.

PPMp was calculated preoperatively using reference EOAi values specific to each valve model and size, indexed to the patient’s body surface area (BSA). The BSA was derived from the patient’s height and weight, and PPMp was extrapolated using the “Valve PPM” application, which incorporates various prosthesis-specific EOAs and gradients.

Informed consent for treatment, data collection, and analysis for scientific purposes was consistently obtained from all patients at the time of hospitalization. The valvular registry at our center (study PRISMA) was approved by the appropriate ethics committee (5973/AO/24).

### 2.1. Magna Ease

The Carpentier-Edwards Perimount Magna Ease valve comprises a cobalt–chromium stent supporting three bovine pericardial leaflets, which undergo the Thermafix tissue treatment process to mitigate calcification. It is designed for supra-annular implantation and is indicated for the treatment of both aortic valve stenosis and regurgitation.

Valve implantation follows a well-established surgical technique [13]. After aortic cross-clamping and cardioplegic arrest, the native valve is excised, and the annular region is meticulously debrided. The annulus is then sized to determine the appropriate prosthesis diameter. Pledget-reinforced, non-everting mattress sutures are placed circumferentially through the aortic annulus and sequentially passed through the sewing ring of the bioprosthesis. The valve is then lowered into position and secured by tying the sutures.

### 2.2. Intuity

The Intuity valve, along with its advanced version, the Intuity Elite, shares key structural similarities with the Magna Ease valve, including pericardial leaflets treated with Thermafix. However, it features an additional balloon-expandable subannular skirt derived from transcatheter valve technology, which enhances both anchoring and sealing. The valve is indicated for aortic stenosis but is contraindicated in cases of aortic regurgitation and endocarditis.

The surgical implantation technique for the Intuity valve has been extensively described in the literature [14]. Following standard aortic cross-clamping and administration of cardioplegia, the diseased native valve leaflets are excised, and the annulus is thoroughly debrided. Three guiding sutures are placed at the nadir of each sinus of Valsalva to facilitate precise positioning of the prosthesis. The valve is then delivered into the aortic annulus, where it is expanded by balloon inflation, ensuring secure fixation within the native annular structure. Once the balloon is deflated and the delivery system is withdrawn, the guiding sutures are tied to reinforce stability.

### 2.3. Inspiris Resilia

The Inspiris Resilia valve is a next-generation stented bioprosthesis constructed from bovine pericardium, and it incorporates two key advancements. First, it features V-Fit technology, which enables controlled and uniform stent expansion during transcatheter valve-in-valve procedures, thereby enhancing long-term structural adaptability. Second, its pericardial leaflets undergo a proprietary integrity preservation process designed to eliminate free aldehydes, a known contributor to tissue calcification, thereby improving durability. The Inspiris Resilia valve is indicated for both aortic valve stenosis and regurgitation.

The surgical implantation of the Inspiris Resilia valve follows a standard supra-annular bioprosthetic replacement technique.

### 2.4. Valve PPM App

Valve PPM is a mobile application designed to assist echocardiography professionals in evaluating the performance of implanted prosthetic heart valves.

The application offers two key functionalities: guidelines for assessing the functionality of an existing valve (Check Valve Function) and support in selecting an optimal valve prior to implantation (Optimal Valve Selector). The algorithms that power the application are based on a comprehensive review of the existing literature on aortic and mitral prosthetic valve assessment.

Upon selecting the specific valve type, patient characteristics (e.g., weight and height), and prosthesis parameters (including peak velocity, mean gradient, VTI prosthesis, VTI LVOT, and LVOTd), Valve PPM provides a diagnostic output. This output informs the user whether the valve is functioning normally, suggests a potential patient–prosthesis mismatch, or indicates possible intrinsic dysfunction of the prosthesis. Additionally, the “Optimal Valve Selector” feature allows users to select a valve position (aortic or mitral) and input patient characteristics. The app then indicates the minimum effective orifice area (EOA) required to avoid patient–prosthesis mismatch. As stated in the app’s additional resources, EOA is calculated by the continuity equation that applies the principle of conservation of mass, where the flow through the LVOT equals the flow through the valve. The app integrates these parameters to compute the EOA as part of its diagnostic output. The continuity equation is widely accepted as the gold standard for EOA calculation in clinical practice, especially in cases of prosthetic valve assessment, due to its robustness in detecting potential patient–prosthesis mismatch (PPM) or valve dysfunction. While the continuity equation is accurate and clinically reliable, it is important to note that its effectiveness depends on precise measurements of the LVOT diameter and VTIs. Errors in measuring the LVOT diameter, which is squared in the equation, can significantly affect the calculated EOA.

### 2.5. Statistical Analysis

Descriptive statistics were reported as median (I–III quartiles) for continuous variables and as absolute numbers (percentages) for categorical variables. The Chi-squared test or Fisher’s exact test, as appropriate, was used to compare the distribution of categorical variables, while the Wilcoxon test was employed to compare the distribution of continuous variables.

The ability of PPMp to predict PPMm was evaluated by calculating sensitivity, specificity, positive predictive value, negative predictive value, and accuracy.

The association of PPMp and PPMm with mortality was evaluated using univariable logistic regression models. Results were reported as Odds Ratio, 95% Confidence Intervals, and *p*-value.

## 3. Results

### 3.1. Baseline

**Figure 1:**
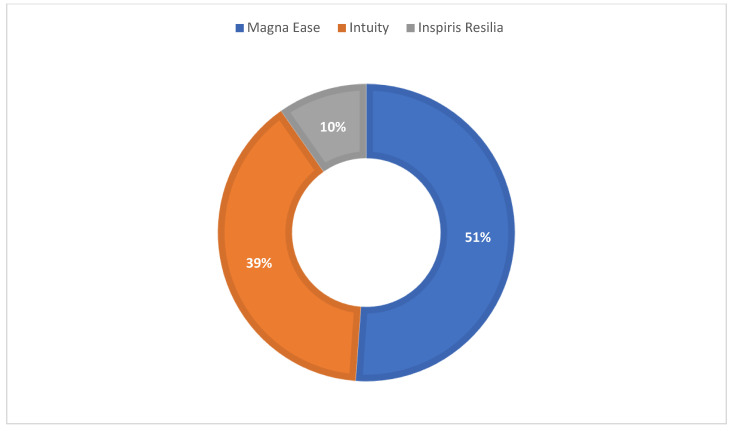
Distribution of the three different bioprostheses in the population.

During the study period, a total of 1323 consecutive patients underwent SAVR at our institution. Of these, 1234 patients received the study devices: 625 patients (50.6%) received the Magna Ease valve, 441 patients (35.7%) received the Intuity valve, and 168 patients (13.6%) received the Inspiris Resilia valve. Complete hemodynamic data were available for 872 patients, who represent the study population. Among them, 446 patients (51.1%) received the Magna Ease valve, 341 patients (39.1%) received the Intuity valve, and 85 patients (9.7%) received the Inspiris Resilia valve (Figure 1).

### 3.2. Measured PPM

Table 1 presents the characteristics of the study population. Prosthesis–patient mismatch (PPMm) was observed in 150 out of 872 patients (17%) in the overall cohort. Patients with PPMm were more frequently implanted with a Magna Ease valve compared with Intuity or Inspiris Resilia valves (65% vs. 11% vs. 23%, *p* < 0.001).

Moreover, when analyzing valve sizes, we found that, as expected, PPMm was more likely to occur in patients who received a size 19 valve (14% vs. 5.4%, *p* < 0.001) or a size 21 valve (26% vs. 22%, *p* < 0.01), irrespective of the type of bioprosthesis implanted.

No significant differences were observed between patients with or without PPMm in terms of height (168 [160, 175] vs. 170 [163, 175], *p* = 0.3), weight (77 [68, 85] vs. 75 [67, 84], *p* = 0.11), and body surface area (BSA) (1.90 [1.71, 1.99] vs. 1.86 [1.74, 2.00], *p* = 0.6).

### 3.3. Predicted PPM

Predicted PPM using the Valve PPM app was found in 165 out of 872 patients (19%). As shown in Table 2, patients with PPMp were shorter (165 [160, 170] vs. 170 [165, 175], *p* < 0.001), thinner (70 [63, 78] vs. 77 [68, 85], *p* < 0.01), and had lower BSA (1.78 [1.65, 1.89] vs. 1.89 [1.76, 2.01], *p* < 0.01).

There were no significant differences according to type of prosthesis (*p* = 0.08); however, PPMp was significantly more evident in the small bioprostheses (size 19: 34% vs. 0.6%, *p* < 0.01; size 21: 61% vs. 14%, *p* < 0.01).

### 3.4. Consistency Between PPMm and PPMp

In 635 out of 872 cases (72.8%), PPMp and PPMm were consistent. Specifically, consistency was observed in 321 out of 446 cases (72%) for the Magna Ease valve, in 256 out of 341 cases (75%) for the Intuity valve, and in 58 out of 85 cases (68.2%) for the Inspiris Resilia valve (Table 3).

Finally, there were no differences between the three different types of bioprosthesis; however, the consistency was significantly lower in the small valve sizes (size 19: 3.6% vs. 16%, *p* < 0.01; size 21: 15% vs. 44%, *p* < 0.01), and it was higher according to AVAi (1.12 [0.98, 1.29] vs. 0.87 [0.78, 1.03], *p* < 0.01).

### 3.5. Accuracy Between PPMm and PPMp

In Table 4 are shown the sensitivity, specificity, positive predicted value, negative predicted value, and accuracy of PPMp overall and for different types of bioprosthesis. After the analyses, it is clear that the methods have a high specificity, both overall and for the types (0.83, 0.86, 0.78, and 0.82), high negative predicted value (0.84, 0.8, 0.93, and 0.79), and a low positive predicted value (0.24, 0.3, 0.19, and 0.14) and low sensitivity.

Clinical associations between mortality and both PPMm and PPMp are reported in Table 5. PPMm had an OR of 1.89 (CI 95% 1.13 to 3.07), *p* = 0.012 and PPMp had an OR of 1.01 (CI 95% 0.57 to 1.71), *p* = 0.96.

## 4. Discussion

The main findings of our research indicate that the consistency between measured patient–prosthesis mismatch (PPMm) and predicted patient–prosthesis mismatch (PPMp) was suboptimal, with no significant differences observed across the different valve types. However, the discordance between PPMm and PPMp was more pronounced in the smaller valve sizes. In cases where smaller valves are implanted, caution should be exercised when relying on PPMp to predict postoperative outcomes, as the risk of unexpected PPMm increases. Neither PPMm nor PPMp was associated with mortality in our cohort, a finding that contrasts with previous reports linking PPM to worse survival outcomes [15].

Despite the lack of association with mortality, accurate PPM prediction remains critical due to its established impact on valve durability and other clinical outcomes, such as heart failure and exercise tolerance. Our findings underscore the importance of a comprehensive understanding of the hemodynamic performance of each valve type, particularly in patients receiving small valve prostheses, where PPM prediction is more complex and less reliable.

Preoperative PPMp prediction tools, such as the Valve PPM app, can aid decision making but should not replace intraoperative judgment or other diagnostic measures. In particular, for smaller valve sizes, PPMp prediction may be limited, and alternative strategies may be necessary to reduce the risk of postoperative PPM. Such strategies include annular enlargement or the use of prosthetic valves designed to minimize transvalvular gradients.

In our study, the Intuity valve demonstrated a lower incidence of PPMm, likely due to its unique design, including the absence of pledgets and the presence of a subannular skirt that opens the left ventricular outflow tract, resulting in a larger effective orifice area (EOA) and lower transvalvular gradients [16]. Previous studies have shown that this valve provides improved hemodynamic performance, making it an optimal choice for patients with small aortic annuli, where annular enlargement may not be feasible.

It is well established that PPMm, based on the indexed effective orifice area (EOAi), is susceptible to measurement inaccuracies and variability in echocardiographic assessments.

Challenges in accurately measuring the left ventricular outflow tract (LVOT) area are a major contributor to the variability seen with PPMm. Minor errors in measuring the LVOT diameter, which is squared in the continuity equation, can lead to substantial inaccuracies in the calculated EOA. However, discrepancies can also stem from the predicted EOA (PPMp) itself. The PPMp is derived using standardized reference values based on valve type, size, and manufacturer specifications, which may not account for patient-specific factors such as hemodynamic conditions, valve seating, or minor anatomical variations post-implantation [17]. These standardized reference EOAs provide an average estimate and may not always reflect the true in vivo performance of the prosthetic valve in a given patient, thereby contributing to some of the inconsistencies observed. We will revise the discussion to better highlight the potential contribution of variability in predicted EOAs to the observed discrepancies.

Computed tomography (CT) or three-dimensional echocardiography may provide more accurate preoperative measurements of the LVOT and aortic annulus, potentially reducing the risk of underestimating the EOA and, consequently, PPMm. The adoption of these advanced imaging techniques could help bridge the gap between preoperative predictions and postoperative measurements of PPM.

The suboptimal consistency between PPMm and PPMp, particularly in smaller valves, raises important clinical and scientific questions. Although PPMp prediction provides a useful framework for guiding surgical decision making, its limitations in certain clinical settings, such as in patients with small aortic annuli, must be acknowledged. This highlights the importance of ongoing clinical vigilance and the need for personalized surgical strategies.

In smaller valves, where PPM risk is heightened, valve design and surgical technique play a pivotal role in minimizing transvalvular gradients and improving hemodynamic performance. Our findings suggest that the Intuity valve, with its specific design advantages, may be an optimal choice in patients where annular enlargement is not feasible, offering a lower risk of postoperative PPMm. These results are also confirmed by other studies already present in the literature, in which the use of sutureless valves [18], particularly in frail patients where annular enlargement may carry a high risk, has proven effective in reducing the incidence of PPM [19].

However, long-term studies are required to assess whether these early hemodynamic advantages translate into improved patient outcomes and valve durability.

Furthermore, the limitations of current preoperative tools like the Valve PPM app—despite its utility—should be considered. While the app can rapidly provide insights into the risk of PPMm, its potential for false positives means that it should be used in conjunction with other diagnostic strategies, such as CT-based annular measurements, to improve preoperative decision making.

The discrepancy between PPMm and PPMp also suggests that new imaging techniques and technological advances, such as the use of artificial intelligence to integrate patient-specific data, may enhance PPM prediction accuracy. Investigating the role of valve-in-valve procedures in managing PPM in small valves could also provide valuable insights for the future.

The importance of assessing PPM is also linked to the increasingly widespread use of TAVI. As reported by Takagi et al. [20] in their review, the incidence of PPM in TAVI procedures is around 35%, and it is not associated with worse outcomes. Recent guidelines also recommend TAVI as a therapeutic option for patients with a low BSA in whom there is a high probability of developing PPM [4]. New studies have aimed to compare PPM incidence between TAVI and SAVR: Alnajar et al. [21], in their review, consider not only patients undergoing TAVI or SAVR as the first choice but also those undergoing redo SAVR and valve-in-valve TAVI. Their review highlights that PPM outcomes after SAVR vary more widely than after TAVI, ranging from 8% to 80% in SAVR and from 24% to 35% in TAVI. The incidence of severe PPM following redo SAVR ranges from 2% to 9%, while following valve-in-valve TAVI, it ranges from 14% to 33%. In conclusion, the choice between one procedure or the other should be made in a selective manner based on the patient’s characteristics in order to ensure the best possible treatment for each patient.

## 5. Conclusions

The use of PPMp is a helpful strategy for reducing the incidence of PPMm, but it remains suboptimal, particularly in smaller valve sizes. A thorough understanding of the specific characteristics of each prosthetic valve is essential to ensure the best possible choice for individual patients.

To further minimize the risk of PPM, annular enlargement to accommodate a larger valve size should be considered whenever feasible, as this approach may improve postoperative hemodynamics and patient outcomes. Additionally, the introduction of advanced imaging techniques and personalized patient assessments may further refine PPM prediction and improve overall clinical outcomes.

The retrospective nature of our study introduces inherent limitations, especially with respect to variability in echocardiographic assessments across different laboratories. Nevertheless, our study is strengthened by the inclusion of a large patient cohort and the comparison of widely used bioprosthetic valves. Future research should focus on long-term hemodynamic data to fully understand the implications of PPM on valve durability and patient survival as well as the role of emerging technologies in PPM management.

## Figures and Tables

**Table 1:** Baseline characteristics of PPMm.

Characteristic	No PPMm (N = 722)	Yes PPMm (N = 150)	*p*-Value
Height (cm)	170 (163, 175)	168 (160, 175)	0.3
Weight (kg)	75 (67, 84)	77 (68, 85)	0.11
BSA (m^2^)	1.86 (1.74, 2.00)	1.90 (1.71, 1.99)	0.6
Prosthesis type			<0.001
Magna Ease	348 (48%)	98 (65%)	
Intuity	306 (42%)	35 (23%)	
Inspiris Resilia	68 (9.4%)	17 (11%)	
Prosthesis size			<0.001
19	39 (5.4%)	21 (14%)	
21	159 (22%)	39 (26%)	
23	238 (33%)	47 (31%)	
25	201 (28%)	34 (23%)	
27	85 (12%)	9 (6.0%)	
Postop AVAi (cm^2^/m^2^)	1.11 (0.99, 1.28)	0.77 (0.69, 0.81)	<0.001
Postop AVA (cm^2^)	2.6 (2.0, 9.8)	1.5 (1.3, 1.9)	<0.001
EOA (cm^2^)	1.80 (1.40, 2.10)	1.80 (1.40, 2.00)	0.009

BSA: body surface area; AVAi: aortic valve area index; AVA: aortic valve area; EOA: effective orifice area.

**Table 2:** Baseline characteristics of PPMp.

Characteristic	No PPMp (N = 707)	Yes PPMp (N = 165)	*p*-Value
Height (cm)	170 (165, 175)	165 (160, 170)	<0.001
Weight (kg)	77 (68, 85)	70 (63, 78)	<0.001
BSA (m^2^)	1.89 (1.76, 2.01)	1.78 (1.65, 1.89)	<0.001
Prosthesis type			0.008
Magna Ease	376 (53%)	69 (42%)	
Intuity	72 (10%)	14 (8.5%)	
Inspiris Resilia	259 (37%)	82 (50%)	
Prosthesis size			<0.001
19	5 (1%)	56 (34%)	
21	98 (14%)	100 (61%)	
23	275 (39%)	9 (5.5%)	
25	235 (33%)	0 (0%)	
27	94 (13%)	0 (0%)	
Postop AVAi (cm^2^/m^2^)	1.09 (0.92, 1.27)	0.98 (0.86, 1.09)	<0.001
Postop AVA (cm^2^)	2.4 (1.8, 9.5)	3.0 (1.7, 8.7)	0.3
EOA (cm^2^)	1.80 (1.70, 2.10)	1.30 (1.20, 1.40)	<0.001

BSA: body surface area; AVAi: aortic valve area index; AVA: aortic valve area; EOA: effective orifice area.

**Table 3:** Consistency between PPMp and PPMm.

Characteristic	No Consistency (N = 237)	Yes Consistency (N = 635)	*p*-Value
Height (cm)	167 (160, 172)	170 (164, 175)	<0.001
Weight (kg)	75 (64, 83)	75 (68, 85)	0.2
BSA (m^2^)	1.83 (1.68, 1.96)	1.87 (1.75, 2.00)	0.010
Prosthesis type			0.4
Magna Ease	124 (52%)	321 (51%)	
Intuity	28 (12%)	58 (9.1%)	
Inspiris Resilia	85 (36%)	256 (40%)	
Prosthesis size			<0.001
19	38 (16%)	23 (3.6%)	
21	105 (44%)	93 (15%)	
23	51 (22%)	233 (37%)	
25	34 (14%)	201 (32%)	
27	9 (4%)	85 (13%)	
Postop AVAi (cm^2^/m^2^)	0.87 (0.78, 1.03)	1.12 (0.98, 1.29)	<0.001
Postop AVA (cm^2^)	1.9 (1.5, 7.9)	2.6 (2.0, 9.8)	<0.001
EOA (cm^2^)	1.80 (1.40, 2.10)	1.40 (1.30, 1.80)	0.016

BSA: body surface area; AVAi: aortic valve area index; AVA: aortic valve area; EOA: effective orifice area.

**Table 4:** Accuracy table.

PPMp	Overall	Magna Ease	Intuity	Inspiris Resilia
Sensitivity	0.26	0.21	0.45	0.11
Specificity	0.83	0.86	0.78	0.82
Positive Predicted Value	0.24	0.3	0.19	0.14
Negative Predicted Value	0.84	0.8	0.93	0.79
Accuracy	0.73	0.72	0.75	0.68

**Table 5:** Association between mortality and PPMm and PPMp.

Characteristics	OR (95% CI)	*p*-Value
PPMm	1.89 (1.13 to 3.07)	0.012
PPMp	1.01 (0.57 to 1.71)	0.96

## Data Availability

No new data were created or analyzed in this study. Data sharing is not applicable to this article.

## Abbreviations

PPM	Prosthesis–patient mismatch
SAVR	Surgical aortic valve replacement
AS	Aortic Stenosis
PPMp	Predicted prosthesis–patient mismatch
PPMm	Measured prosthesis–patient mismatch
EOAi	Effective orifice area index
BSA	Body surface area
LVH	Left ventricular hypertrophy
ID	Internal diameter
VTI	Velocity time integral
LVOT	Left Ventricle outflow tract
CT	Computed tomography
OD	Odds Ratio
CI	Confidence Interval
AVAi	Aortic valve area index
